# Absence of the dolichol synthesis gene DHRSX leads to N-glycosylation defects in Lec5 and Lec9 Chinese hamster ovary cells

**DOI:** 10.1016/j.jbc.2024.107875

**Published:** 2024-10-10

**Authors:** Takfarinas Kentache, Charlotte R. Althoff, Francesco Caligiore, Erika Souche, Céline Schulz, Julie Graff, Eline Pieters, Pamela Stanley, Joseph N. Contessa, Emile Van Schaftingen, Gert Matthijs, François Foulquier, Guido T. Bommer, Matthew P. Wilson

**Affiliations:** 1 Metabolic Research Group, de Duve Institute & WELRI, Université Catholique de Louvain, Brussels, Belgium; 2 Laboratory for Molecular Diagnosis, Center for Human Genetics, KU Leuven, Leuven, Belgium; 3 Univ. Lille, CNRS, UMR 8576 – UGSF - Unité de Glycobiologie Structurale et Fonctionnelle, Lille, France; 4 Laboratory for Cytogenetics and Genome Research, Department of Human Genetics, KU Leuven, Leuven, Belgium; 5 Department of Cell Biology, Albert Einstein College of Medicine, New York, New York; 6 Department of Therapeutic Radiology, Yale School of Medicine, New Haven, Connecticut; 7 Department of Pharmacology, Yale School of Medicine, New Haven, Connecticut

**Keywords:** glycosylation, N-linked glycosylation, glycobiology, glycoprotein biosynthesis, glycoconjugate, isoprenoid, lipid synthesis, dolichol, polyprenol, polyprenal, CHO glycosylation mutants

## Abstract

Glycosylation-deficient Chinese hamster ovary cell lines have been instrumental in the discovery of N-glycosylation machinery. Yet, the molecular causes of the glycosylation defects in the Lec5 and Lec9 mutants have been elusive, even though for both cell lines a defect in dolichol formation from polyprenol was previously established. We recently found that dolichol synthesis from polyprenol occurs in three steps consisting of the conversion of polyprenol to polyprenal by DHRSX, the reduction of polyprenal to dolichal by SRD5A3, and the reduction of dolichal to dolichol, again by DHRSX. This led us to investigate defective dolichol synthesis in Lec5 and Lec9 cells. Both cell lines showed increased levels of polyprenol and its derivatives, concomitant with decreased levels of dolichol and derivatives, but no change in polyprenal levels, suggesting DHRSX deficiency. Accordingly, N-glycan synthesis and changes in polyisoprenoid levels were corrected by complementation with human DHRSX but not with SRD5A3. Furthermore, the typical polyprenol dehydrogenase and dolichal reductase activities of DHRSX were absent in membrane preparations derived from Lec5 and Lec9 cells, while the reduction of polyprenal to dolichal, catalyzed by SRD5A3, was unaffected. Long-read whole genome sequencing of Lec5 and Lec9 cells did not reveal mutations in the ORF of *SRD5A3*, but the genomic region containing *DHRSX* was absent. Lastly, we established the sequence of Chinese hamster DHRSX and validated that this protein has similar kinetic properties to the human enzyme. Our work therefore identifies the basis of the dolichol synthesis defect in Chinese hamster ovary Lec5 and Lec9 cells.

Glycosylation mutants affecting numerous proteins involved in the glycosylation process have been isolated in Chinese hamster ovary (CHO) cells mainly by selection for resistance to toxic lectins. More than 40 different complementation groups have been characterized in this way, and in many cases, mutations in glycosylation pathway genes have been identified (1, 2). However, the genetic defects in Lec5 (or B211) and Lec9 mutants have not been defined. These cell lines are characterized by reduced N-glycan synthesis and an alteration in the abundance of specific N-glycan subtypes, due to a defect in the conversion of polyprenol to dolichol (3, 4, 5, 6, 7, 8).

Dolichol plays an important role in various types of glycosylation as a carrier of monosaccharides (Dol-P-glucose and Dol-P-mannose) and of the lipid-linked oligosaccharide (LLO) that is transferred *en bloc* onto the N-X-S/T/C motif of proteins (where N is asparagine, X is any amino acid except proline, and S/T/C is serine, threonine or, rarely, cysteine) during N-glycosylation. Polyprenol, the precursor of dolichol, is formed *via* the mevalonate pathway and differs from dolichol only by the presence of a double bond between carbons 2 and 3 of the terminal isoprene unit (9). When polyprenol is used as a monosaccharide or oligosaccharide carrier, this difference considerably alters the activity of several enzymes that usually act on different dolichol derivatives in the context of glycosylation (10, 11, 12, 13, 14).

Though the lack of conversion of polyprenol to dolichol in Lec5 and Lec9 cells is well established, the mechanism of this defect is still unknown (6, 8, 15, 16). Polyprenol conversion to dolichol was, in 2010, proposed to involve an NADPH-dependent reductase (17). The demonstration of the role of the *SRD5A3* gene product in the conversion of polyprenol to dolichol led to the assumption that SRD5A3 was the long sought polyprenol reductase. Surprisingly, this finding did not lead to the identification of the defect in Lec5 and Lec9 cells, including in our hands (unpublished Sanger sequencing data).

The widely held view that SRD5A3 is a polyprenol reductase has been challenged recently by our group (18). We demonstrated that the conversion of polyprenol to dolichol is a three-step process, involving polyprenal and dolichal as intermediates, and in which SRD5A3 is a polypren*a*l (not a polypren*o*l) reductase. Another enzyme, encoded by the *DHRSX* gene, catalyzes the two other steps, that is, the oxidation of polyprenol to polyprenal and the final reduction of dolichal to dolichol (Fig. 1*A*). In this work, we show that the *DHRSX* gene is deleted in Lec5 and Lec9 cells and that both their dolichol synthesis and N-glycan defects are rescued by human DHSRX.

**Figure 1:**
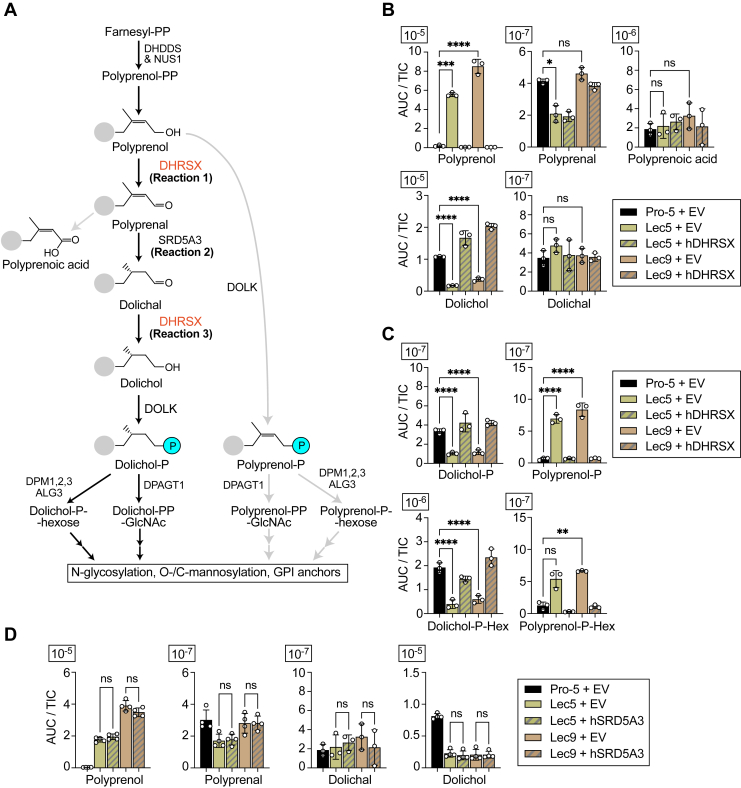
**Accumulation of polyisoprenoid species in Lec5/9 cells resembles that in *Homo sapiens* DHRSX deficiency.***A*, mammalian dolichol biosynthesis pathway. *B*, polyisoprenoid species in Pro-5 (control), Lec5, and Lec9 CHO cells complemented or not with human DHRSX. See Fig. S1*A* for all isoprenyl chain lengths. *C*, phosphoisoprenoid and hexose phosphoisoprenoid species in Pro-5 (control), Lec5, and Lec9 CHO cells complemented or not with human DHRSX. See Fig. S1*B* for all isoprenyl chain lengths. *D*, polyisoprenoid species in Pro-5 (control), Lec5, and Lec9 CHO cells complemented or not with human SRD5A3. See Fig. S1*C* for all isoprenyl chain lengths. *B*–*D*, data represent area under the curve (AUC) normalized to total ion count (TIC) of species with 19 isoprenyl units (means ± SD; n = 3 biological replicates). For statistical analysis, an ordinary one-way ANOVA followed by a Tukey’s multiple comparisons test was used; ns, ∗∗, ∗∗∗, and ∗∗∗∗ represent not significant, *p* < 0.01, *p* < 0.001, and *p* < 0.0001, respectively.

## Results

### Accumulation of polyisoprenoid species in Lec5 and Lec9 cells resembles that in *Homo sapiens* DHRSX deficiency

Studies on human cell lines show that DHRSX deficiency leads to a decrease in dolichol and an increase in polyprenol and similar changes in glycosylation to those previously observed with Lec5 and Lec9 cells (18). In particular, previous work on the glycosylation defect in CHO Lec5 and Lec9 cells showed that the conversion of polyprenol to dolichol is deficient (16, 19). As a result of this, N-glycan synthesis in Lec5 and Lec9 cells utilizes polyprenol-phosphate rather than dolichol-phosphate species, leading to perturbation in the synthesis and transfer of the LLO (Fig. 1*A*) (18).

These changes resemble those we found when inactivating SRD5A3 or DHRSX in human cell lines. In both cases, we observed a strong accumulation of polyprenol-derived lipids alongside a reduced level in dolichol-derived lipids. Biochemically, the main feature that distinguishes DHRSX from SRD5A3 deficient cells is that only the latter accumulate the intermediate polyprenal and its carboxyl derivative, polyprenoic acid (Fig. 1*A*) (18).

To identify the defect causing Lec5 and Lec9 phenotypes, we analyzed isoprenoids in these cells compared to their parental cell line, Pro-5. As previously observed for SRD5A3- and DHRSX-deficient cells, we observed accumulation of polyprenol (28- and 43-fold for Lec5 and Lec9, respectively), polyprenol-phosphate (11- and 13-fold), and polyprenol-phosphohexose (4- and 5-fold) and a decrease in dolichol (6- and 3-fold), dolichol-phosphate (both by 3-fold), and dolichol-phosphohexose (5- and 3-fold) (Fig. 1, *B* and *C*, see Fig. S1*A* for all measured lengths; 17–20).

However, Lec5 and Lec9 cells did not show a significant accumulation of polyprenal and polyprenoic acid, as is commonly observed in SRD5A3 deficiency. This suggested that the defective enzyme was DHRSX. Accordingly, complementation of Lec5 or Lec9 cells with *H. sapiens* (h) DHRSX restored polyisoprenoid species levels similar to those observed in control Pro-5 cells, whereas complementation with *H. sapiens* (h) SRD5A3 had no effect (Fig. 1, *B–D*, see Fig. S1, *A* and *B* for all measured lengths; 17–20).

### DHRSX activity is absent in Lec5 and Lec9 cells, but SRD5A3 activity is unaffected

DHRSX is a quite unique dehydrogenase/reductase in that it catalyzes two distinct reactions: i) the conversion of polyprenol to polyprenal (Reaction 1, Fig. 1*A*) and ii) that of dolichal to dolichol (Reaction 3, Fig. 1*A*). Accordingly, it also has similar affinities for NAD(H) and NADP(H). By contrast, SRD5A3 is a NADPH-specific reductase that converts polyprenal to dolichal (Reaction 2, Fig. 1*A*). These enzymatic activities can be readily measured in human cell extracts such as HAP1 and lymphoblastoid cells (18).

**Figure 2:**
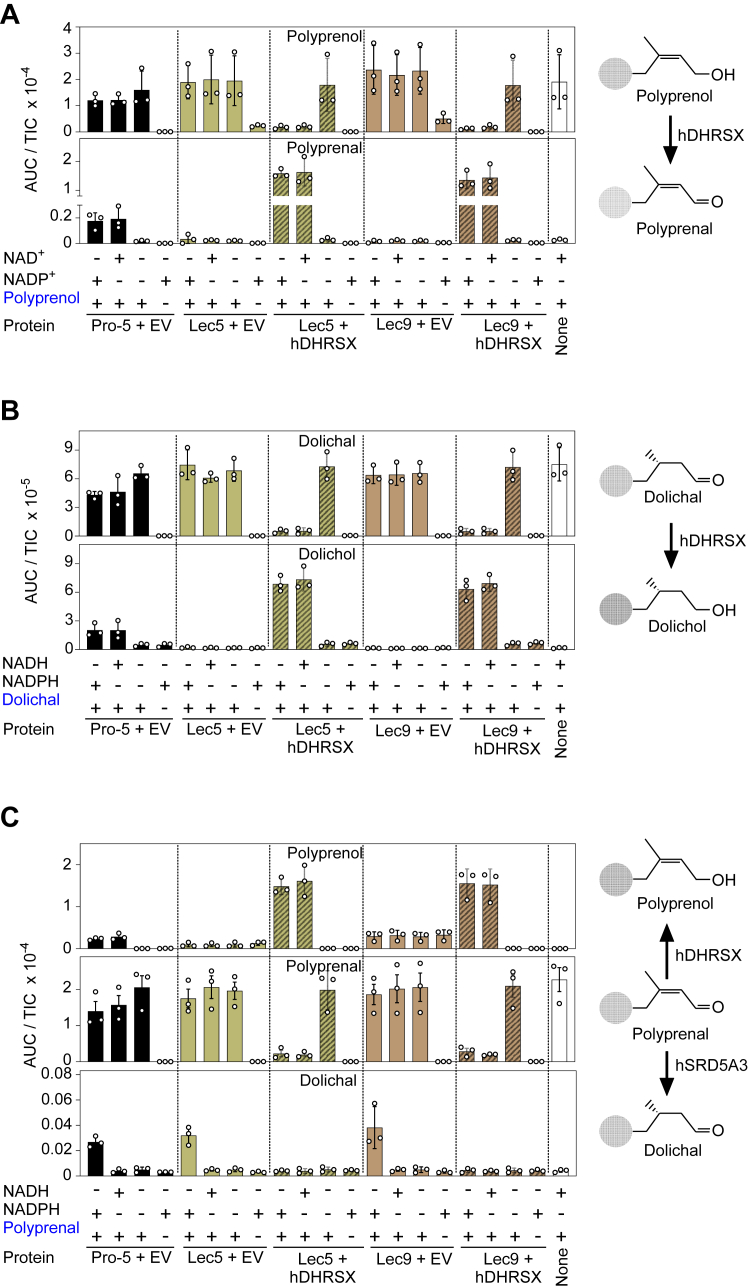
**DHRSX activites are absent in Lec5 and Lec9 cells, but SRD5A3 activity is unaffected.***A*, failure of Lec5 and Lec9 CHO cells to form polyprenal from polyprenol. Polyprenal formation from polyprenol was measured after incubation of 5 μg/ml of a 13 to 21 isoprene unit–containing polyprenol mixture with 5 mM NADP^+^ or NAD^+^ and 1 mg/ml membrane extracts for 2 h at 37 °C. Measurements are based on the formation of polyprenal with 18 isoprene units (means ± SEM, n = 3). Data is TIC-normalized AUC (means ± SD; n = 3 biological replicates). *B*, Lec5 and Lec9 CHO cells lack dolichal reductase activity. Dolichal-18 and dolichol-18 were measured in reactions containing 1 mg/ml CHO membrane proteins, 5 μg/ml dolichal mixture, and 5 mM NAD(P)H for 2 h at 37 °C. Data is TIC-normalized AUC (means ± SD; n = 3 biological replicates). *C*, detection of NADPH-dependent polyprenal reductase activity mediated by SRD5A3 in Lec5 and Lec9 CHO cells. SRD5A3 activity was determined by measuring dolichal formation after incubation of 5 μg/ml of a polyprenal mixture with 5 mM NAD(P)H and 1 mg/ml membrane preparations for 2 h at 37 °C. The reverse reaction of polyprenol dehydrogenase mediated by DHRSX (see (*A*)) was detected as well. The panel shows only species with 18 isoprene units. Data is TIC-normalized AUC (means ± SD; n = 3 biological replicates).

To confirm the identity of the enzymatic defect, we assayed these activities in membrane preparations derived from Pro-5, Lec5, and Lec9 cells, including those expressing hDHRSX. Using polyprenol and NAD^+^ or NADP^+^ as substrates, we clearly detected the formation of polyprenal in membranes derived from WT cells, but not from Lec5 or Lec9 cells (Fig. 2*A*). As expected, expression of hDHRSX in Lec5 and Lec9 cells increased the formation of polyprenal even beyond that observed in Pro-5 cells.

We also obtained similar results when we assessed the reduction of dolichal to dolichol, the second enzymatic function of DHRSX (Fig. 2*B*). Dolichol formation was clear in Pro-5 cells but absent in Lec5 and Lec9 cells. Again, expression of hDHRSX strongly increased dolichol formation. The absence of both activities characteristic for DHRSX indicates that the Chinese hamster ortholog of DHRSX is deficient in Lec5 and Lec9 cells, and that, similarly to the human enzyme, Chinese hamster (ch) DHRSX is able to catalyze both the conversion of polyprenol to polyprenal and the conversion of dolichal to dolichol, with no apparent specificity for NADP(H) or NAD(H).

To ensure that the SRD5A3 activity was not affected in Lec5 or Lec9 cells, we incubated the same cell membrane preparations with polyprenal and either NADH or NADPH. We noted a comparable formation of dolichal in Pro-5, Lec5, and Lec9 cells, in the presence of NADPH, but not with NADH. This indicated that SRD5A3 activity was normal in Lec5 and Lec9 cells (Fig. 2*C*).

Surprisingly, the formation of dolichal from polyprenal seemed to be abolished in cells overexpressing DHRSX. This is explained by the fact that DHRSX catalyzes, *in vitro*, the reduction of polyprenal to polyprenol in the presence of either NADH or NADPH, leading to the strong increase of polyprenol formation in membrane preparations from cell lines overexpressing DHRSX (Fig. 2*C*). Consequently, the substrate of SRD5A3, polyprenal, will be rapidly depleted leading to the formation of polyprenol at the expense of dolichal.

Taken together, these enzymatic assays indicated that DHRSX activity is absent in Lec5 and Lec9 cells and that there is no deficiency of SRD5A3 activity in either cell line.

### The glycosylation defect in Lec5 and Lec9 cells is rescued by the expression of human DHRSX

We used two complementary methods to assess glycosylation status in Lec5 and Lec9 cells: in depth characterization of an artificial glycosylation marker protein *via* Western blotting and an unbiased proteomic characterization of microsomal proteins.

**Figure 3:**
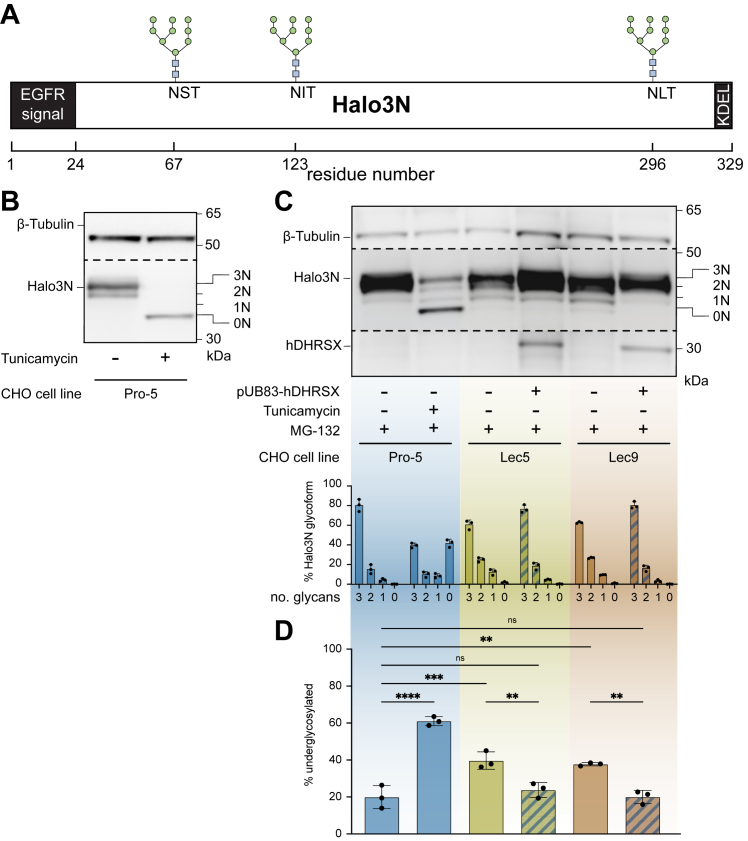
**The glycosylation defect in Lec5 and Lec9 cells is rescued by the expression of human DHRSX.***A*, the Halo3N glycomarker protein with N-glycosylation sites indicated. *B*, Halo3N is a sensitive marker of N-glycosylation status in Pro-5 CHO cells. SDS-PAGE analysis shows that tunicamycin treatment (1 μg/ml) for 24 h abolishes all N-glycan attachment to Halo3N and signal derived from probing with a HaloTag antibody is condensed to a single band (0N). Blot was also probed with a β-tubulin antibody as a protein loading control. *C*, Lec5 and Lec9 CHO cells have deficient N-glycan attachment, rescued by the expression of pUB83-hDHRSX. Probing with a HaloTag antibody revealed that treatment with a proteasomal inhibitor (MG-132, 10 μmol/L, 24 h) allows improved resolution of intermediate (2N, 1N, and 0N) Halo3N glycosylation states in tunicamycin (1 μg/ml for 24 h) treated Pro-5 cells. This facilitated their quantification in untreated Pro-5, Lec5, and Lec9 cells. hDHRSX expression was confirmed by reprobing with a DHRSX antibody and the blot was also probed with a β-tubulin antibody as a protein loading control (means ± SD; n = 3 biological replicates). See Fig. S2 for Ponceau S total protein stain of (*C*). *D*, underglycosylation of the Halo3N reporter protein in Lec5 and Lec9 cells is restored to levels in Pro-5 control cells by the expression of hDHRSX. % underglycosylated represents the signal intensity of the 0N, 1N, and 2N glycosylation states combined, after probing with a HaloTag antibody, compared to overall signal including the 3N glycosylation state. ns,∗∗, ∗∗∗, and ∗∗∗∗ represent not significant, *p* < 0.01, *p* < 0.001, and *p* < 0.0001, respectively. For statistical analysis, an ordinary one-way ANOVA followed by a Tukey’s multiple comparisons test was used (means ± SD; n = 3 biological replicates).

Human DHRSX-deficient cell lines showed reduced glycosylation of LAMP2 (18). Unfortunately, antibodies directed at the human protein did not allow us to detect this protein in CHO cells (data not shown). We therefore used an artificial protein, Halo3N, containing three well-defined N-glycosylation sites. Halo3N is a derivative of the HaloTag (20) engineered to contain three N-glycosylation sites, an EGFR signal sequence, and a KDEL ER-retention signal, allowing the quantification of N-glycosylation site occupancy based on different migration profiles by SDS-PAGE analysis (Fig. 3*A*) (21).

After stable expression of Halo3N in Pro-5, Lec5, and Lec9 cells, we first validated that the migration profile of this protein was indeed dependent on the glycosylation status in CHO cells. To this end, we analyzed lysates from Pro-5 cells cultured in the presence and absence of tunicamycin, an inhibitor of DPAGT1, the enzyme that transfers *N*-acetylglucosamine-1-phosphate onto dolichol phosphate in the early stages of lipid-linked oligosaccharide biosynthesis. Halo3N has four possible glycosylation states, carrying 3, 2, 1, or no N-glycans (hereafter 3N, 2N, 1N, and 0N). Most of the signal in samples from untreated Pro-5 cells was concentrated in a single band, presumably corresponding to the fully glycosylated form (3N), whereas samples from Tunicamycin-treated Pro-5 cells showed a single band with a faster migration in SDS-PAGE, presumably corresponding to the unglycosylated form (0N; Fig. 3*B*).

Next, we compared Pro-5 with Lec5 and Lec9 cells and cell lines in which human DHRSX was overexpressed. These experiments were performed in the presence of the proteasome inhibitor MG-132 to limit potential degradation of hypoglycosylated proteins. In Pro-5 cells treated with tunicamycin, this allowed us to detect four bands for Halo3N, presumably corresponding to the glycoforms 0N, 1N, 2N, and 3N. Halo3N in untreated Pro-5 CHO cells was observed to be 79% fully glycosylated (3N), with 16% and 5% of the 2N and 1N forms, respectively (Fig. 3, *C* and *D*). Both Lec5 and Lec9 cells showed a reduction in the fully glycosylated form and a concomitant significant increase in the hypoglycosylated forms. Both changes were rescued by the expression of hDHRSX, leading to Halo3N glycosylation states undistinguishable from that of Pro-5 cells.

**Figure 4:**
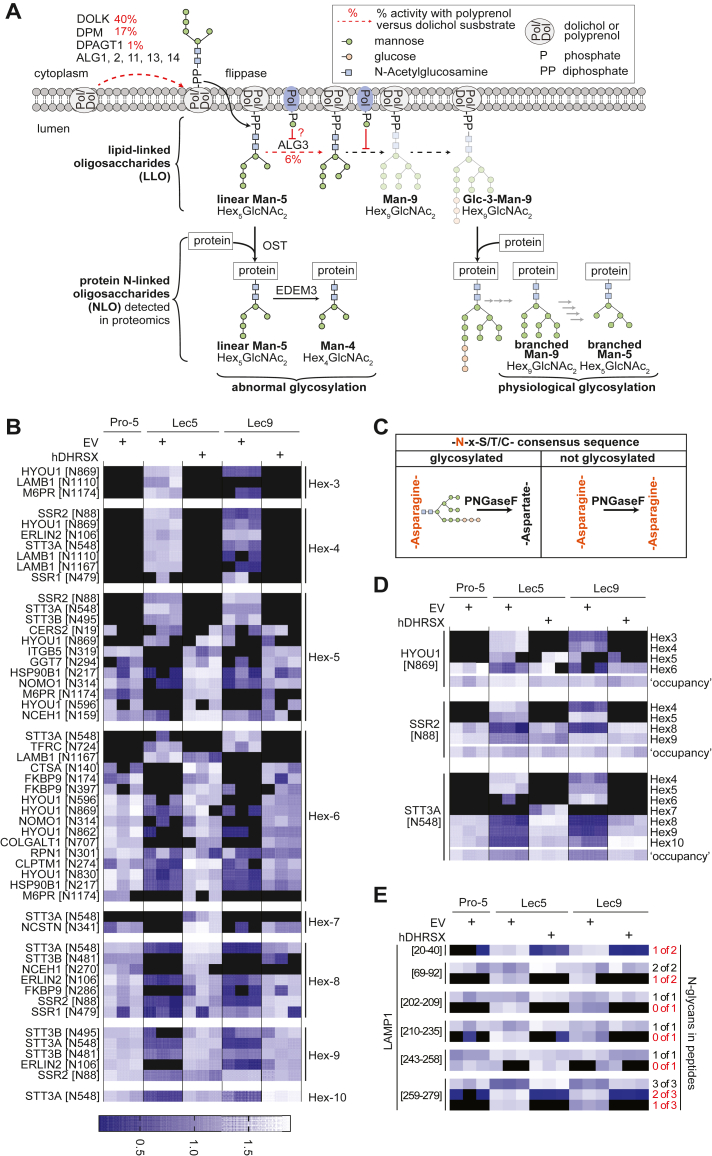
**Lec5 and Lec9 cells show glycan structures that disappear upon expression of human DHRSX.***A*, schematic representation of the formation of N-linked glycans under physiological conditions and when dolichol is largely replaced by polyprenol (18). Note that the maturation beyond linear Man5-GlcNAc2 LLO is inefficient in the latter situation. *B*, quantification of the indicated glycopeptides in the membrane fractions of Pro-5, Lec5, and Lec9 cell lines. Each cell line was transduced either with a lentiviral vector containing an empty vector (EV), or, in case of Lec5 and Lec9, a vector inducing the expression of human DHRSX (hDHRSX). The heatmap represent the abundance of different N-glycosylation sites, regrouped by glycosylation types from Hex3-GlcNAc2 to Hex10-GlcNAc2. Signals obtained in three biological replicates were log2-transformed and are shown relative to the 70th percentile within each row to facilitate visualization. High values are presented in *white* and low values in *dark blue* (according to the color scale in the figure), whereas *black* cells indicate missing values (see Table S1 for non-normalized data). *C*, schematic of the PNGase F reaction on N-glycosylation sites. Hydrolytic removal of the attached glycans forms an aspartate residue, which can be distinguished from the asparagine residue present in the nonglycosylated peptide. *D*, detailed comparison of the different glycosylation states at selected glycosites, presented as in (*B*). This panel also includes data on the abundance of PNGase F–treated samples (‘occupancy’). *E*, abundance of peptides derived from the protein LAMP1 carrying 0, 1, 2, or 3 N-glycans. Peptides were quantified by nano-LC-MS/MS after treatment with PNGaseF, which converts glycan-carrying asparagine residues to aspartates. The numbers on the *left* indicate the location of the peptides within LAMP1. The numbers on the *right* indicate how many of the possible glycosylation sites were occupied. Hypoglycosylated forms are highlighted in *red*. In *B*, *D*, and *E*, a brighter colour indicates higher signals, whereas *dark blue* indicates lower signals for the indicated peptides (as indicated in the color scale in the figure). *Black* indicates that a peptide was not detected.

We also explored the glycosylation state of Pro-5, Lec5, and Lec9 cells *via* a proteomic approach. Inactivation of DHRSX in human HAP1 cells leads to a shift from dolichol to polyprenol as the LLO/monosaccharide carrier and to an increase in the transfer of immature glycans onto proteins (Fig. 4*A*) (18). We detected several Hexose(Hex)3-GlcNAc2, Hex4-GlcNAc2, Hex5-GlcNAc2, and even Hex6-GlcNAc2 glycans in Lec5 and Lec9 cells, which were absent in Pro-5 cells or when we re-expressed hDHRSX (Fig. 4*B* and Table S1). Extension of the linear Man5-GlcNAc2 glycan is much less efficient when the LLO is assembled on polyprenol pyrophosphate as opposed to dolichol pyrophosphate (Fig. 4*A*) (12). Thus, the Hex5-GlcNAc2 peptides increased in Lec5 and Lec9 cells are likely the result of the transfer of linear Man5-GlcNAc2 onto proteins.

Linear Man5-GlcNAc2 can be subjected to two enzymatic processes: On the one hand, a glucosylation/deglucosylation cycle plays a role in permitting proper protein folding. Accordingly, some of the Hex6-GlcNAc2 glycans can be formed by the transfer of glucose residues to Man5-GlcNAc2 using ER glucosyltransferases, leading to Glc1-Man5-GlcNAc2 (22). On the other hand, demannosylation *via* the enzyme EDEM3 can target unfolded and hypoglycosylated proteins to ER-associated protein degradation (23). Accordingly, the Hex4-GlcNAc2 and Hex3-GlcNAc2 glycans appearing in Lec5 and Lec9 cells likely correspond to Man4-GlcNAc2 and Man3-GlcNAc2 glycans (21).

Conversely, a distinct subset of Hex5-GlcNAc2 glycans and several Hex6-GlcNAc2, Hex7-GlcNAc2, and Hex8-GlcNAc2 glycans were lost in Lec5 and Lec9 cells but reappeared upon expression of human DHRSX, likely representing intermediates in the physiological N-glycosylation pathway, including branched (as opposed to linear) Man5-GlcNAc2 glycans. In Lec5 and Lec9 cells, the shift towards immature N-glycans at the expense of the fully extended N-glycans was also apparent when we analyzed individual peptides for which several different glycosylation states were observed (Fig. 4*D*). Even though comprehensive data are available only for a limited number of proteins, our findings document a clear change in glycan structures in Lec5 and Lec9 cells.

To explore whether these changes affect overall N-glycosylation site occupancy, we treated peptides with PNGaseF before proteomic analysis. This removes N-glycans, resulting in an aspartate residue which can be distinguished from the asparagine to which the glycan was attached (Fig. 4*C*). Changes in the occupancy were rather subtle or absent for the peptides presented in panels B and D (Fig. 4, *C* and *D*; ‘occupancy’). Yet, when focusing on several peptides of the highly glycosylated protein LAMP1, we observed considerable heterogeneity between glycosylation sites (Fig. 4*E*). Hypoglycosylated or unglycosylated forms appeared for all peptides, but only in one instance, this was paralleled by a reduction in the abundance of the fully glycosylated form (amino acids 259–279, Fig. 4*E*). This suggested that as well as N-glycans being absent altogether, in many cases, mature glycans might be replaced by immature glycans, agreeing with previous glycoproteomic studies on human LLO biosynthesis enzyme deficiencies (24, 25).

Overall, we document a quantitative change evidenced by reduced Halo3N glycosylation and a site-dependent reduction in glycosylation site occupancy of LAMP1 and other endogenous glycoproteins, as well as a structural change evidenced by the attachment of immature N-glycans to glycoproteins in Lec5 and Lec9 cells. Both these changes were rescued by the expression of hDHRSX.

### CHO cells have a functional DHRSX ortholog that is absent in Lec5 and Lec9 CHO cells

A number of lectin-resistant CHO glycosylation mutant cell lines have either deletions or point mutations inactivating genes important for N-glycosylation (1). We therefore investigated whether a genetic mechanism was causing the DHRSX deficiency in Lec5 and Lec9 cells. Initially, attempts to determine the genomic and RNA-derived coding sequences of *DHRSXvia* Sanger sequencing in Lec5/9 cells were unsuccessful. A partial *DHRSX* sequence was acquired in Pro-5 cells, but efforts were hampered by sequence differences, as compared to those of available *Cricetulus griseus* reference sequences (*e.g.*, CHO-K1 CriGri_1.0, GenBank: GCA_000223135.1; CriGri-PICRH-1.0, GenBank: GCA_003668045.2; CHOK1S_HZDv1, GenBank: GCA_900186095.1).

To overcome this problem, we carried out Oxford Nanopore Technology (ONT) long-read whole genome sequencing using genomic DNA extracted from Pro-5, Lec5, and Lec9 CHO cells and performed *de novo* genome assembly for each cell line. This led to full-length assemblies that were of similar quality to those previously published (26) (Tables 1 and S2). In Pro-5 cells, *DHRSX* was detected towards the end of a contig of 385 kbp (contig 8156; Figs.S3 and 5*D*). We were unable to identify reads pertaining to *DHRSX* in Lec5 or Lec9 cells.

**Table 1:** Parameters of *de novo* assembly in this study compared to available *C. griseus* (PICRH) and CHO-K1 (CHOZN v2.3) reference genomes

Assembly	This study	This study	This study	PICRH (2020)	CHOZN v2.3 (2022)
DNA Source	WT Pro-5 CHO	Lec5 CHO	Lec9 CHO	*C. griseus* tissues	CHO-K1 cells
Assembly length (Gb)	2.39	2.35	2.35	2.37	2.30
Contig N50	13,782,288	14,039,095	13,096,259	274,391,693	43,523,667
Contig N90	1,667,153	1,803,015	1,796,527	127,255,434	549
Contig L50	48	47	51	4	17
No. of contigs	3013	2119	2241	647	1,634,314

N50: Length of the shortest contig at 50% of assembly length (*i.e.*, 50% of assembly is in contigs of this length or longer); N90: length of the shortest contig at 90% of assembly length (*i.e.*, 90% of assembly is in contigs of this length or longer); L50: the minimum number of contigs that, combined, make up 50% of the assembly length.

**Figure 5:**
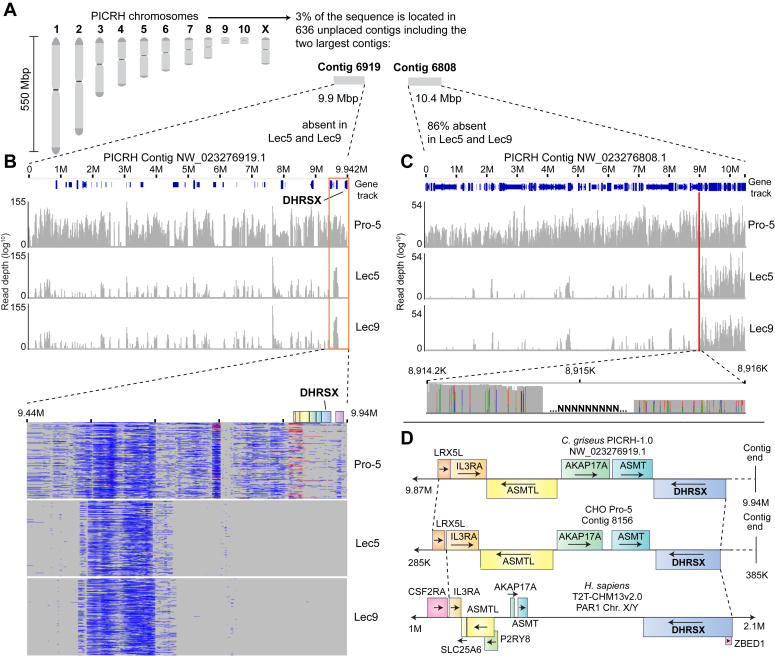
**The *DHRSX* gene is absent in Lec5 and Lec9 CHO cells.***A*, the Chinese hamster chromosomes. *B*, *upper* panel: The contig containing *DHRSX* is devoid of consistent reads in Lec5 and Lec9 CHO cells but present in parental Pro-5 cells. Chart shows the coverage (range 0–155, log^10^ scale) of reads recovered from ONT long-read whole genome sequencing of Pro-5, Lec5, and Lec9 CHO cells, mapped to contig NW_023276919.1 of the PICRH-1.0 (GenBank: GCA_003668045.2) *C. griseus* genome assembly. Coverage calculation (*via* igvtools count) and visualization performed using Integrative Genomics Viewer (IGV) 2.17.1 (46). Gene track = GCF_003668045.3. *Lower panel*: reads from ONT sequencing of Pro-5, Lec5, and Lec9 CHO cells mapped to the terminal (9.44–9.94 M) region of contig NW_023276919.1 of the PICRH-1.0 CHO genome. Visualization performed using Ribbon (47). *C*, *upper* panel: The first 8915 Kbp of the unplaced contig NW_023276808.1 are absent in Lec5 and Lec9 cells. Chart shows the coverage (range 0–54, log^10^ scale) of reads recovered from ONT long-read whole genome sequencing of Pro-5, Lec5, and Lec9 CHO cells, mapped to contig NW_023276808.1 of the PICRH-1.0 (GenBank: GCA_003668045.2) *C. griseus* genome assembly. *Red line* indicates position upstream of which reads are almost completely absent in Lec5 and Lec9 cells. Coverage calculation (*via* igvtools count) and visualization performed using Integrative Genomics Viewer (IGV) 2.17.1 (49). Gene track = GCF_003668045.3. *Lower panel*: the gap of ±600 bp containing N’s at approximately 8915 Kbp in Pro-5 reads mapped to PICRH NW_023276808.1 indicates that the reads upstream and downstream of this location are not contiguous. Visualization performed using Integrative Genomics Viewer (IGV) 2.17.1. *D*, conservation of synteny in the genomic region surrounding *DHRSX* in the PICRH-1.0 *C. griseus* genome, contig 8156 derived from the *de novo* genomic assembly of Pro-5 CHO cells in this study, and *H. sapiens* T2T-CHM13v2.0. Note extended scale in *H. sapiens* (20× that of CHO genomes).

Next, we mapped our ONT long-reads to the CriGri-PICRH-1.0 genome (subsequently referred to as PICRH). This is the Chinese hamster genome with the most complete chromosomal information, with 97% of the genome placed on 11 ‘mega-scaffolds’ pertaining to the Chinese hamster chromosomes (Fig. 5*A*) (27). Coverage of all 11 characterized chromosomes was normal in both Lec5 and Lec9 cells, when compared to Pro-5 cells (Table S3). The same was found when, instead of mapping ONT long-reads directly to the PICRH reference genome, we used the consensus reference sequences created by *de novo* assembly of each respective genome (Tables 2 and S4).

**Table 2:** Mean coverage of consensus sequence derived from *de novo* assembly of CHO Pro-5, Lec5, and Lec9 genomes when mapped to the chromosomes of the PICRH reference genome

PICRH chromosome	Length (bp)	Pro-5 coverage (%)	Lec5 coverage (%)	Lec9 coverage (%)
1	550,089,852	100	100	100
2	461,620,117	100	100	100
3	282,827,514	100	100	100
4	231,097,868	100	100	100
5	193,770,019	099	099	099
6	155,611,870	100	099	099
7	134,359,064	100	100	099
8	99,554,469	113	111	111
9	28,505,831	103	107	102
10	32,558,357	101	101	100
X	127,255,434	103	102	103

See Table S4 for data from all PICRH contigs.

In the PICRH genome, *DHRSX* is located close to the end of an unplaced contig of approximately 10 Mbp (NW_023276919.1). The mapping of Pro-5 reads to this contig resulted in consistent coverage. However, our long-read data sets from Lec5 and Lec9 cells left this contig empty, except for reads in repetitive regions that are unlikely to be representative of real coverage or alignment. This indicated that this region is entirely absent in the mutant cell lines (Fig. 5*B*), in turn suggesting that both Lec5 and Lec9 cells carried a large deletion of at least 10 Mbp.

Several observations indicate that the deletion is in fact larger: First, approximately 86% of an additional unplaced contig of 10.5 Mbp, NW_023276808.1, was absent in Lec5 and Lec9 cells (Fig. 5*C*). While the abrupt end of read alignments might give the impression of a distinct breakpoint in Lec5 and Lec9 cells, this transition occurs in a region that contains unidentified sequences (‘NNN’ in Fig. 5*C*) and is not spanned by any reads, suggesting that the assembly of this contig is incorrect. Second, Lec5 and Lec9 cells lack reads in many other unplaced contigs (see Tables S2 and S3), suggesting that the deletion is likely even larger. Given that no major differences could be identified in the gross coverage of any contig between Lec5 and Lec9 cells, deletions in both cells were likely spanning a very similar region that exceeds 19 Mbp.

To attempt a better localization of *DHRSX* compared to a deletion breakpoint or telomere, we explored the synteny of this region between PICRH contig NW_023276919.1, contig 8156 in our *de novo* assembly of the Pro-5 cell genome, and corresponding human sequences. Indeed, the organization and directionality of gene transcripts in the terminal regions of these two contigs are conserved extremely well between hamster assemblies (Fig. 5*D*). The same is largely true in *H. sapiens*, though, intriguingly, the intergenic and intronic sequences were greatly expanded (∼20×) compared to those in PICRH and Pro-5 cells. Thus, in Lec5 and Lec9 cells, *DHRSX* and its genetic neighborhood (containing *LRX5L, IL3RA, ASMTL, AKAP17A*, and *ASMT*) is entirely absent (Fig. 5, *B* and *D*).

The closest gene to *H. sapiens DHRSX* in the centromeric direction is *CD99* (alongside its pseudogene *CD99P1*). *CD99* is found close to the start of another short PICRH contig of 120kb (NW_023277415.1), also missing in Lec5 and Lec9 cells. In our mapping, overlapping reads in Pro-5 cells between NW_023276919.1 and this contig indicate that they are also contiguous in *C. griseus*, but genes further along this contig do not correspond to their human orthologs (*XG, GYG2, ARSD, ARSE, ARSL*). Given the unplaced nature of the hamster contigs, we were not able to determine whether *DHRSX* was located close to the chromosomal end or close to the end of the deletion.

**Figure 6:**
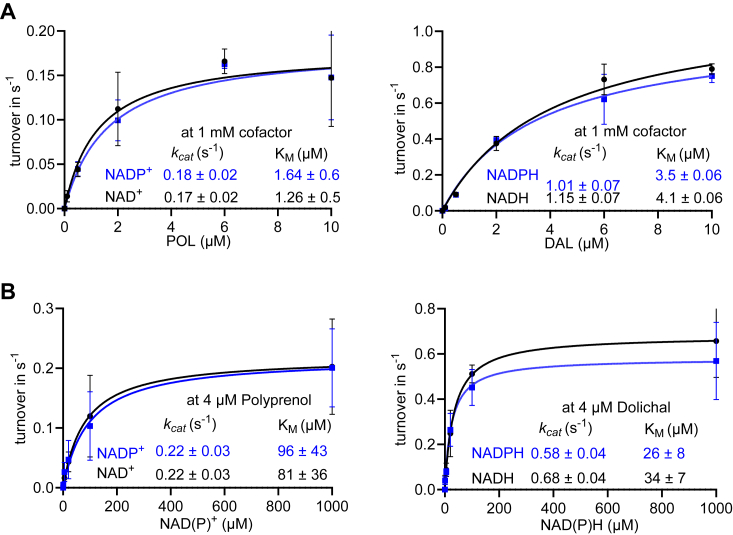
**Chinese hamster DHRSX is functional with similar kinetic parameters to the human ortholog.***A*, for polyprenol and dolichal: Kinetic parameters were determined by measuring polyprenal or dolichol formation after incubation of the indicated concentrations of polyprenol or dolichal, respectively. Assays were performed in the presence of 1 mmol/L of the indicated cofactors and 0.01 μmol/L recombinant chDHRSX for 5 min at 37 °C. Data are turnover rates based on the formation of polyprenal-18 or dolichol-18 (means ± SD; n = 3 biological replicates). *B*, for NAD(P)^+^ and NAD(P)H: identical incubation was performed with variable nucleotide concentrations in the presence of 4 μmol/L polyprenol or dolichal and measuring the formation of polyprenal or dolichol, respectively. Data are turnover rates based on the formation of polyprenal-18 or dolichol-18 (means ± SD; n = 3 biological replicates).

Finally, to confirm that chDHRSX was active and catalyzed the same activities as hDHRSX, we overexpressed and purified recombinant chDHRSX using the amino acid sequence predicted from whole genome long-read sequencing of Pro-5 CHO cells (Fig. S3). When assessing polyprenol dehydrogenase and dolichal reductase activities, we observed kinetic parameters comparable with those observed for hDHRSX (Fig. 6 and Table 3).

**Table 3:** Kinetic properties of chDHRSX determined in this study and those of hDHRSX previously reported (See ref. (18))

Kinetic property	Polyprenol	Dolichal	NAD^+^	NADP^+^	NADH	NADPH
K_cat_ (s^−1^)						
chDHRSX (This study)	0.17 ± 0.02	1.01 ± 0.07	0.22 ± 0.03	0.22 ± 0.03	0.68 ± 0.04	0.58 ± 0.04
hDHRSX (See ref. (18))	0.44 ± 0.05	1.00 ± 0.13	0.16 ± 0.03	0.18 ± 0.01	0.62 ± 0.08	0.65 ± 0.05
K_M_ (μM)						
chDHRSX (This study)	1.3 ± 0.5	3.5 ± 0.1	81 ± 36	96 ± 43	34 ± 07	26 ± 08
hDHRSX (See ref. (18))	5.7 ± 1.2	2.2 ± 1.3	66 ± 43	42 ± 11	45 ± 24	40 ± 12

## Discussion

### Solving an old riddle

Lec5 and Lec9 cells are characterized by deficient conversion of polyprenol to dolichol (3, 4, 5, 6, 7, 8) but the genetic basis of this phenotype was not identified. With the discovery of the role of SRD5A3 in the conversion of polyprenol to dolichol (17), a likely candidate for the molecular defect in Lec5 and Lec9 cells was revealed. However, it took the discovery of the role of DHRSX in the formation of dolichol (18), alongside the reassignment of the function of SRD5A3, to allow us to identify, here, the biochemical and genetic bases of the dolichol defect in Lec5 and Lec9 cells.

### A comparable large deletion comprising *DHRSX* causes the glycosylation defect in Lec5 and Lec9 cells

Lec5 and Lec9 cells had already been postulated to have the same gene defect since Lec5/Lec9 hybrid cells produce polyprenol rather than dolichol (16, 28). However, earlier experiments showed Lec5/Lec9 hybrids are sensitive to ricin, indicative of complementation and a different genetic basis for each phenotype (29). A compensatory mechanism that allows ricin resistance in hybrids might exist, *via* a similar mechanism to that observed in certain cells from DHRSX-CDG patients (18). Alternatively, ricin resistance in CHO cells can arise from mutations affecting protein synthesis (30), which may be present in either Lec5 or Lec9 cells.

Nevertheless, we show here that both Lec5 and Lec9 have a large deletion of at least 19 Mbp, leading to a loss of *DHRSX*, alongside many other genes, in contigs NW_023276919.1 and NW_023276808.1 of the PICRH *C. griseus* genome assembly. It appears therefore that the *DHRSX* gene is prone to inactivation by dramatic rearrangements, consistent with the fact that *DHRSX* RNA was not identified in transcriptomics studies on Lec5 cells by Lu *et al.* (31). Given that both cell lines have a comparable deletion, a similar deletion may also have occurred in the several other cell lines that spontaneously arose and that fall in the same complementation group as Lec5 or Lec9 cells (*e.g.*, CHB11-1-3 cells (16)). All these cell lines were selected for their resistance to toxic lectins that bind to N-glycans. Thus, even if this deletion might occur at an extremely low rate (the genomic instability of CHO cells is well documented (32, 33)) and is unfavorable to growth, it likely confers a selective advantage under the significant selective pressure applied by the lectin.

### CHO Lec5 and Lec9 revertants: An ongoing puzzle

The glycosylation phenotype of Lec5 and Lec9 cells is quite puzzling, since spontaneous revertants have been repeatedly observed (1, 29, 31). The large genomic deletion observed in these cell lines excludes the possibility that DHRSX might be reactivated. To account for this easy reversibility, Lu *et al.* suggested that the mutation(s) causing the Lec5 phenotype were affecting the epigenetic regulation of one or more genes; specifically, *via* homeobox genes. Our findings do not exclude this possibility. Conceivably, epigenetic modulation of one of the multiple dehydrogenases belonging to the same family as DHRSX (*e.g.*, RDH11-14, DHRS7, DHRS13, WWOX) could lead to sufficient polyprenol dehydrogenase and dolichal reductase activity to restore sufficient glycosylation. However, the Lec5 revertant RNA-Seq data collected by Lu *et al.* does not show increased expression of any of these genes specifically. Another possibility for reversion might be the occurrence of mutations altering the kinetic properties in one of the many dehydrogenases belonging to the same family as DHRSX and retinol dehydrogenases.

Alternatively, the fact that revertants of CHB11-1-3 (Lec5) cells synthesize normal levels of dolichol but with higher levels of polyprenol (16) suggests that perhaps the polyprenol biosynthesis pathway (derived from the mevalonate/cholesterol biosynthesis pathway) is somehow upregulated in revertants. This might increase polyprenol levels to those high enough that sufficient N-glycosylation is restored, thereby diminishing their lectin-resistant phenotype. However, the opposite of this was identified in the gene expression data of Lu *et al.*, with the expression of mevalonate pathway genes such as 3-hydroxy-3-methylglutaryl-CoA synthase 1, (*Hmgcs1*) downregulated in Lec5 revertants.

It is likely that if another dehydrogenase can replace chDHRSX, it has a higher K_m_ for polyprenol. This situation resembles that in human cells: loss of DHRSX or SRD5A3 in both model cell lines and patient cells such as fibroblast and lymphoblasts does not result in the complete absence of dolichol. In some cases, levels are almost completely normalized, despite persistent polyprenol or polyprenal accumulation (18, 34). This suggests that the defects in either of these enzymes can be compensated at the cellular level by a yet unknown mechanism. Perhaps the compensatory mechanism in Lec5 and Lec9 revertants is, in fact, replicated in human cells, but not enough to prevent disease.

To what extent this type of compensation mechanism occurs widely in different tissues and can attenuate specific symptoms, would certainly be worth investigating. Of note, the defect in the glycosylation of transferrin present in both DHRSX-CDG and SRD5A3-CDG can be absent, or normalize with age (18, 35). Understanding the mechanism of reversion of the defect in CHO cells could also apply to human disease.

## Experimental procedures

For a full list of reagents used in this study, see Key Resources Table.

**Table:** Key Resources Table

Reagent or resource	Source	Identifier
Antibodies/lectins		
DHRSX	Sigma-Aldrich	HPA003035
β-tubulin	Thermo Fisher Scientific	MA516308
HRP-linked anti-rabbit IgG	Bioké	7074S
HRP-linked anti-mouse IgG	Bioké	7076S
HaloTag®	Promega	G9211
Chemicals, reagents, and equipment		
Bovine Serum Albumin (BSA) Fraction V	Sigma	10735086001
Penicillin/Streptomycin Solution (100×)	CAPRICORN Scientific	CP21-4278
Fetal Bovine Serum	Dutcher	S1810
Nitrocellulose membrane	Invitrogen	LC2000
Fetal Bovine Serum	Sigma-Aldrich	98022
Lipofectamine 3000	Thermo Fisher Scientific	15292465
DMEM/F-12	Gibco	21041-025
DMEM/F-12, HEPES	Gibco	31330038
Cytiva HyClone™ Fetal Clone III Serum	Cytiva	12319862
Triton™ X-100	Sigma-Aldrich	T8787
beta-mercaptoethanol	Sigma-Aldrich	M3148
Pageruler Plus Prestained Protein Ladder	Thermo Fisher Scientific	11832124
DMSO	Sigma-Aldrich	317275
Paraformaldehyde	Thermo Fisher Scientific	28908
Mowiol® 4-88	Sigma-Aldrich	81381
RIPA buffer	Custom	N/A
SuperSignal West Pico PLUS Chemiluminescent Substrate	Thermo Fisher Scientific	Cat# 34579
Puromycin dihydrochloride	Gibco	A1113802
G-418 Geneticin Selective Antibiotic	Gibco	10131035
Tunicamycin	Sigma-Aldrich	SML1287
MG-132	VWR	474787
Pyridinium dichromate	Merck life science BV	214698-100G
PBS	Thermo Fisher Scientific	10010023
TBS 10×	Euromedex	ET220
Versene	Gibco	15040033
Methanol	Thermo Fisher Scientific	10606652
Methanol (LC-MS grade)	Biosolve	136878
Chloroform	VWR	22711290
Acetic acid, 99.8% for analysis	Thermo scientific	222140010
Dichloromethane	Merck	1.06050.1000
Chloroform (LC-MS grade)	Biosolve	34806
trimethylsilyl diazomethane (TMSD)	Sigma-Aldrich	362832
NAD^+^ free acid grade II	ROCHE	10127990001
NADH disodium salt	ROCHE	10128023001
NADP^+^ sodium salt hydrate	Sigma-Aldrich	N0505-1G
NADPH tetrasodium salt	ROCHE	10102824001
Dolichol	Avanti polar lipids	9002000
Polyprenol	Avanti polar lipids	9002100
Dolichal	This study	N/A
Polyprenal	Avanti polar lipids (discontinued)	9002200
Phosphatidylcholine	Sigma-Aldrich	P-0378
Phosphatidylethanolamine	Sigma-Aldrich	P-0503
Carbenicillin	VWR	J67159.AD
Gelred nucleic acid stain	Sigma-Aldrich	SCT123
Agilent 6546 ion funnel mass spectrometer	Agilent	N/A
Agilent 1290 HPLC System	Agilent	N/A
Accucore C30 150 × 2.1 mm column	Thermo Fisher Scientific	27826-152130
EASY-Spray 0.075 × 250 mm	Thermo Fisher Scientific	ES902
Trap-column	Thermo Fisher Scientific	Acclaim PepMap100
Isopropopanol (MS-grade)	Biosolve	162678
Acetonitrile	Biosolve	12078
Ammonium formate	Biosolve	19878
Formic acid	Biosolve	232478
iBlot 2 NC mini Stacks	Invitrogen	IB23002
ECL WB substrate	Thermo Fisher Scientific	PIER32106
DNAse I	Sigma Aldrich	10104159001
Trypsin	Gibco	25300096
Tris-buffered saline	Bioké	124985
IPTG	Thermo Fisher Scientific	15529019
Bolt® 4-12% Bis-Tris Plus Gels, 12-well	Thermo Fisher Scientific	15324604
Bolt Transfer Buffer (20×)-1 L	Thermo Fisher Scientific	15256066
20× Bolt® MES SDS Running Buffer (500 ml)	Thermo Fisher Scientific	13266499
LDS sample buffer	Thermo Fisher Scientific	11549166
cOmplete, Mini, EDTA-free Protease	Thermo Fisher Scientific	11836170001
Trypsin	Sigma-Aldrich	T8003-500MG
Trypsin-EDTA 1× in PBS w/o calcium w/o magnesium	Dominique Dutscher	L0940-100
Tween-20	Sigma-Aldrich	P1379
N-Glycosidase F	Roche	11365193001
6× DNA loading dye	Thermo Fisher Scientific	R0611
Polybrene	Merck life science N.V (ex Sigma Aldrich)	TR-1003
Phusion DNA polymerase	Thermo Fisher Scientific	F630S
T4 DNA ligase, 5 U/μL	Thermo Fisher Scientific	EL0014
Trizma base	Merck Life Science BV (ex Sigma Aldrich)	T1503-100g
HEPES	Merck Life Science BV (ex Sigma Aldrich)	H3375-500G
Antipaine	Merck Life Science B.V. (Sigma)	A6191
Leupeptine	Merck Life Science B.V. (Sigma)	L2884
PMSF	Merck Life Science B.V. (Sigma)	10837091001
Phosphate buffered saline PBS TABLETS	Millipore	2810306
PNGase F	NEB	P0704S
S-Trap micro units	Protifi LLC	N/A
HisTrap-HP 1 ml	GE Healthcare	GE29-0510-21
Imidazole	Merck Life Science BV (ex Sigma Aldrich)	56750-500G
Water UPLC/MS - CC/SFC	Biosolve	232141
Sequencing Grade Modified Trypsin (1 × 100 ug)	Promega	V5117
Lys-C endopeptidase	Sopachem NV	125-02543
Phosphoric acid	Merck Life Science BV (ex Sigma Aldrich)	695017-100ML
Sodium dodecyl sulfate SDS	Merck Life Science BV (ex Sigma Aldrich)	62862-1KG
Commercial assays/kits		
Micro BCA Protein Assay Kit	Thermo Fisher Scientific	Cat# 23235
Ligation Sequencing Kit XL V14	ONT	SQL-LSK114-XL
HS Large Fragment 50kb Kit	Agilent Technologies	DNF-464-33
Pierce Quantitative Peptide Assay	Thermo Fisher Scientific	23275
Monarch HMW DNA Extraction Kit	NEB	T3050L
Experimental models: Cell lines/strains		
HEK293T	Gift from Reid Gilmore, Uni. Of Mass.	This paper
BL21 (DE3) *E. Coli*	Thermo Fisher Scientific	10749734
XL1-Blue Competent Cells *E. coli*	Agilent	200249
Oligonucleotides		
SRD5A3_rev_bsrGI ATACAATGTACACTAGAAGGCACAGTCGAGGC	This paper	N/A
SRD5A3_sense_BgIII TTATATAGATCTCCACCATGGCTCCCTGGGCGGAG	This paper	N/A
hDHRSX_rev_bsrGI ATACCATGTACATCACAGGGTCACATCAAGGAC	This paper	N/A
hDHRSX_ sense_BgIII TTATATAGATCTCCACCATGTCGCCATTGTCTGCGG	This paper	N/A
Plasmids and vectors		
pET15b DHRSX-N-His NM_145177.3	This paper	N/A
pET15b chDHRSX-N-His	This paper	N/A
pcDNA3.1 (+) SRD5A3-N-His NM_024592.5	This paper	N/A
pcDNA3-ER-Halo3N	Rinis *et al.* 2018	N/A
pUB83 hSRD5A3-N-His	This paper	N/A
pUB83 hDHRSX-N-His	This paper	N/A
Software and algorithms		
ImageJ 2.14.0	NIH	https://www.imagej.net/ij/
Fiji	NIH	https://doi.org/10.1038/nmeth.2019
Adobe Illustrator	Adobe	https://www.adobe.com
Interactive Genomics Viewer	Broad Institute	https://software.broadinstitute.org/software/igv/
Ugene	Unipro	https://www.ugene.net/
Graphpad Prism 10.1	Dotmatics	https://www.graphpad.com
Mass Hunter Quantitative Analysis	Agilent	https://www.agilent.com/en/product/software-informatics/mass-spectrometry-software/data-analysis/quantitative-analysis
Mass Hunter Qualitative Analysis	Agilent	https://www.agilent.com/en/product/software-informatics/mass-spectrometry-software/data-analysis/quantitative-analysis
Proteome Discoverer 2.5 SP1	Thermo Fisher Scientific	https://www.thermofisher.com/be/en/home/industrial/mass-spectrometry/liquid-chromatography-mass-spectrometry-lc-ms/lc-ms-software/multi-omics-data-analysis
MiniKNOW Core 5.4.3	ONT	https://nanoporetech.com/news/news-introducing-new-minknow-app
NanoPlot 1.41.0	PyPI	https://pypi.org/project/NanoPlot/
Flye assembler 2.9.2	Bioconda	https://anaconda.org/bioconda/flye
Mosdepth 0.3.5	Bioconda	https://anaconda.org/bioconda/mosdepth
racon 1.5.0	ILRI Research Computing	https://hpc.ilri.cgiar.org/racon-software
QUAST 5.2.0	QUAST	https://quast.sourceforge.net/docs/manual.html
Integrative Genomics Viewer (IGV) 2.17.1	Integrative Genomics Viewer	https://igv.org/
Protparam tool	expasy	https://web.expasy.org/protparam/

### CHO cell culture

CHO cells were cultured in DMEM/F12 (Gibco, 31330-038) supplemented with 10% FBS (Clone III, HyClones) and antibiotics streptomycin (100 μg/ml), penicillin (100 U/ml), and amphotericin (0.5 μg/ml) at 37 °C under 5% CO_2_. Cells were passaged using trypsin (Gibco, 25300096) and versene (Gibco, 15040066). Regular tests for *mycoplasma* were performed by PCR (SouthernBiotech, 1310001).

### Plasmid constructions

The recombinant plasmid pET15b chDHRSX-N-His, pET15b hDHRSX-N-His (NM_145177.3), and pcDNA3.1(+) hSRD5A3-N-His (NM_024592.5) were purchased from GenScript Biotech and sequences were checked by Sanger sequencing. The primer sets hDHRSX_sense_BglII_HIS/hDHRSX_rev_BsrGI or SRD5A3_sense_BglII_HIS/SRD5A3_rev_bsrGI, respectively, were used to PCR-amplify hDHRSX or hSRD5A3 from pET15b hDHRSX-N-His and pcDNA3.1(+) hSRD5A3-N-His using Phusion DNA polymerase, 2 U/μl (F-530S, Thermo Fisher scientific) following the recommendations of the manufacturer (see Key Resources Table). BgIII and BsrGI restriction enzymes were used for cloning into pUB83. T4 DNA ligase, 5 U/μl (EL0014, Thermo Fisher scientific) was used for the insertion of hDHRSX or hSRD5A3 in pUB83 predigested by the same restriction enzymes. pUB83 is a lentiviral expression vector based on the pLVX-PURO (Clontech) plasmid containing a CMV promoter. It contains ampicillin and puromycin resistance cassettes for bacterial and eukaryotic cell selection, respectively. Sequence of recombinant vector pUB83 hDHRSX-N-His and pUB83 hSRD5A3-N-His were verified by Sanger sequencing. The pcDNA3-ER-Halo3N construct has been described previously (21).

### Recombinant lentiviruses production and infection of CHO cells

Recombinant lentiviruses were produced in HEK293T cells as previously described (36) using recombinant vector pUB83 hDHRSX-N-His, pUB83 hSRD5A3-N-His, or empty pUB83. Viruses were then used to infect Pro-5, Lec5, or Lec9 cells. CHO cells were grown in six well plates to reach 60% confluence on the day of infection in DMEM/F12. The medium was replaced by DMEM/F12 containing diluted recombinant lentiviruses (lentivirus: DMEM 1:4 V/V) in the presence of 4 μg/ml polybrene. After 24 h of growth, the medium was replaced by virus-free DMEM/F12 containing 30 μg/ml puromycin.

### Isoprenoid species and preparation of dolichal

Dolichol (# 9002000), polyprenol (# 9002100), and polyprenal (# 9002200) were purchased from Avanti Polar Lipids. Dolichal was synthesized by oxidizing dolichol in the presence of pyridinium dichromate (37) (# 214698-100G, Merck life science BV): 300 µl of 1 mg/ml dolichol in chloroform was dried down and resuspended in 200 µl of dichloromethane containing 10 mg/ml of pyridinium dichromate. After shaking at 1000 rpm for 15 min, the preparation was incubated overnight at 20 °C. The mixture was centrifuged, and the supernatant was transferred to a new tube. Several cycles of back-extractions were performed by using 200 µl methanol/water (5:3) (MS-grade, Biosolve) to remove pyridinium dichromate until the preparation became translucent. The quality of dolichal synthesis was assessed by LC-MS, showing a high yield of conversion (>95%). Dolichal was stored at −80 °C for future enzymatic assays. All isoprenoid solutions represent mixtures with 13 to 21 isoprene units (with 18 being the most abundant species). Molar concentrations were calculated based on the distribution of chain lengths. Thus, a 5 μg/ml solution corresponds to approximately 4 μM.

### Sample preparation and extraction of metabolites from CHO cells

The medium was removed, plates were rapidly washed with ice-cold water, and plunged in liquid nitrogen to quench metabolic activity. The frozen dishes were placed on dry ice, and 500 μl ice-cold methanol was immediately added, followed by 300 μl of ice-cold water. The cells were scraped and collected in 2 ml tubes containing 1 ml of chloroform (MS-grade, Biosolve). Tubes were vigorously mixed at 2000 rpm for 20 min at 4 °C, followed by a centrifugation at 13,200 rpm for 30 min at 4 °C. The lower layer, containing hydrophobic metabolites, was dried down under a gentle stream of nitrogen and resuspended in 100 μl of methanol/isopropanol (1:1) before LC–MS analysis.

### Dimethylation of isoprenoid phosphates using trimethylsilyl diazomethane

The dimethylation of dolichol phosphate and polyprenol phosphate was performed as described in Kale *et al.* (38): The organic fraction containing isoprenoid species was completely dried and resuspended in 200 μl of dichloromethane:methanol (6.5:5.2, v/v). Ten microliters of 2 mol/L trimethylsilyl diazomethane was added and incubated for 40 min at room temperature. The incubation was halted by adding 1 μl of glacial acetic acid. Samples were then dried and dissolved in methanol:isopropanol 1:1 (V/V) for LC-MS analysis.

### Extraction of membrane proteins from CHO cells

Confluent 10 cm plates were washed with ice-cold PBS and cells were collected in 0.5 ml/plate of lysis buffer (25 mmol/L Hepes pH 7.5, 2 μg/ml antipain and leupeptin, 0.5 mmol/L PMSF, and 10% glycerol) by scraping plates. Cells were then subjected to two cycles of freezing-thawing in liquid nitrogen, treated with DNAse I (0.1 mg/ml in 10 mmol/L MgSO_4_) for 15 min, and centrifuged (13,200 rpm for 15 min at 4 °C) to recover a membrane fraction in the pellet. The resulting pellet was washed twice by centrifugation as above with the same volume of lysis buffer, and the resulting pellet was resuspended in the initial volume of lysis buffer. This preparation was aliquoted and kept at −80 °C.

### Measurement of DHRSX or SRD5A3 activities in the membrane protein extracts of CHO cells

Enzymatic activity measurements were performed in buffer containing 10 mmol/L Hepes, pH 8.0, 10 mmol/L KCl, 0.3% Triton X-100, 0.5 mmol/L β-mercaptoethanol, 1% phosphatidylcholine (PC), and 0.2% phosphatidylethanolamine (PE). To assess dehydrogenase or reductase activities, 5 μg/ml of polyprenol, polyprenal, or dolichal were used in the presence of 5 mmol/L of NAD(H)^+^ or NADP(H)^+^. First, 5 μl of a of 50 μg/ml solution of the respective polyisoprenoid mixtures in chloroform was added into an empty tube and dried under a stream of nitrogen. After that, 5 μl of 10× buffer [100 mmol/L Hepes, pH 8.0, 100 mmol/L KCl, 3% Triton X-100, 0.5 mmol/L β-mercaptoethanol, 10% PC, and 2% PE] were added to the tube. Five microliters of NAD(P)^+^ or NAD(P)H (50 mM) was then added. The incubations were started by adding protein extracts at a final concentration of 1 mg/ml in a total volume of 50 μl. Incubation was performed at 37 °C for 2 h. Samples were analyzed by LC-MS after methanol-chloroform extraction of analytes.

### Analysis of recombinant chDHRSX activity on polyprenol and dolichal

To determine kinetic properties of recombinant chDHRSX, measurements were performed in a buffer containing 10 mmol/L Hepes pH 8.0, 10 mmol/L KCl, 0.5 mmol/L β-mercaptoethanol, 1% PC, and 0.2% PE. 4 μmol/L of lipid substrates were used in the presence of 0, 1, 5, 20, 100, or 1000 μmol/L of NAD(P)^++^ or NAD(P)H to investigate kinetic properties of cofactors. For the ones of polyprenol and dolichal: 0, 0.1, 0.5, 2, 6, or 10 μmol/L of lipids were used in the presence of 1 mmol/L of NAD(H). After a preheating at 37 °C, the assay was started by the addition of 0.01 μmol/L final concentration of recombinant chDHRSX and carried out in a total volume of 50 μl at 37 °C and 400 rpm. After 5 minutes of incubation, assays were immediately quenched by methanol-chloroform extraction. Nonlinear curve fitting with GraphPad Prism 10.1 was used assuming Michaelis–Menten kinetics.

### Sample preparation and extraction of metabolites from enzymatic assays

After the end of each respective incubation, 450 μl of ice-cold methanol/water (5:3) was added into the tube followed by 550 μl of ice-cold chloroform. The mixture was vigorously mixed at 2000 rpm for 20 min and at 4 °C, followed by a centrifugation at 13,200 rpm for 10 min at 4 °C. The lower layer containing hydrophobic metabolites was dried down under a gentle stream of nitrogen and resuspended in 50 μl methanol/isopropanol (1:1) before LC–MS analysis.

### Stable expression of pcDNA3-Halo3N-ER

Pro-5, Lec5, or Lec9 CHO cells already overexpressing empty pUB83 or hDHRSX pUB83 were reverse transfected using the Lipofectamine 3000 reagent (Thermo Fisher Scientific) with 0.5 μg of the pcDNA3-Halo3N-ER plasmid into 6-well plates containing DMEM/F-12 and 30 μg/ml puromycin. After 24 h, medium was swapped with that also containing 1 mg/ml G-418 (Sigma, A1720) for selection. For treatment with tunicamycin (Sigma, SML1287) and MG-132 (VWR, 474787), cells were plated on 6-well plates, and after 24 h, medium was exchanged for that also containing 1 μg/ml tunicamycin and/or 10 μmol/L MG-132. Cells were cultured for a further 24 h before collection of lysates for immunoblot analysis.

### SDS-PAGE and Western blot

For immunoblotting, protein lysates from CHO cells were prepared by lysing cells in RIPA buffer (10 mmol/L Tris–HCl [pH 7.4], 150 mmol/L NaCl, 0.5% sodium deoxycholate, 0.1% SDS, and 1× cOmplete protease inhibitor cocktail [Sigma Aldrich]) at 4 °C, passing 5 times through a 27G needle, incubating for 30 min followed by centrifugation at 4 °C (15,000*g*, 30 min). Protein concentration was determined with the Pierce BCA protein assay kit (Thermo Fisher Scientific, 23225). Lysates were separated by SDS-PAGE and blotted onto a nitrocellulose membrane (Invitrogen, LC2000). Blocking was performed in 2 to 5% milk or bovine serum albumin (98022, Sigma-aldrich) with the relevant primary and secondary antibodies. Washing was performed with 1× Tris-buffered saline solution (12498S, Bioké) with 0.1% Tween-20 (P1379, Sigma-Aldrich). Signal detection was performed by chemiluminescence using an Amersham ImageQuant 800 imager (Cytiva) and quantification using ImageJ 2.14.0. Primary antibodies used were anti-DHRSX (Sigma, HPA003035), anti-β-tubulin (Thermo Fisher Scientific, MA516308), anti-HaloTag (Promega, G9211) and secondary antibodies were HRP-linked anti-rabbit (7074S) or mouse (7076S) IgG (Bioké). Secondary antibodies and the monoclonal antibodies recognizing β-tubulin and HaloTag are well established, very specific antibodies validated by their manufacturers. The polyclonal anti-DHRSX antibody (Sigma, HPA003035) has been shown previously to specifically detect native DHRSX levels in human cells and in both human and yeast cells overexpressing DHRSX and purified DHRSX protein (18). For a full list of antibodies used in this study, see Key Resources Table.

### LC–MS analysis of metabolites

LC–MS analysis of organic fractions obtained from cells (or enzymatic assays) was carried out using a method adapted from that used by Dewulf *et al.* 2019 (39). Briefly, 5 μl of sample was injected and subjected to reverse phase chromatography with an Accucore C30 150 × 2.1 mm column (ref 27826-152130, Thermo Fisher Scientific), operated at 45 °C on an Agilent 1290 HPLC system. The flow rate was constant at 0.2 ml/min using mobile phase A (60% acetonitrile and 40% water, 10 mmol/L ammonium formate, and 0.1% formic acid) and B (90% isopropanol, 10% acetonitrile, 10 mmol/L ammonium formate, and 0.1% formic acid (Biosolve)). An Agilent 6546 mass spectrometer was used in the positive or negative ionization modes with an electrospray ionization (voltage 3500 V, Nozzle voltage 1000 V, sheath gas 350 °C at 11 L/min, nebulizer pressure 35 psi, and drying gas 300 °C at 8 L/min). Starting from 5 min onwards, one spectrum encompassing a range of 69 to 1700 *m/z* was acquired per second, generated from 10,772 transients. The mass spectrometer was operated in positive polarity for the detection of dolichal, polyprenal, dolichol, polyprenol, dimethylated dolichol-P, and dimethylated polyprenol-P. For the elution, the solvent gradient was as follows: 0 to 5 min at 90% B; 5 to 33 min from 90 to 97% B; 33 to 34 min from 97 to 99% B; 34 to 35 min from 99 to 90% B. The negative polarity was used to detect and measure polyprenoic acid, dolichol-P, polyprenol-P, dolichol-P-hexose, and polyprenol-P-hexose. The elution gradient consisted of the following: 0 to 3 min at 30% B; 3 to 8 min from 30 to 43% B; 8 to 9 min from 43 to 50% B; 9 to 18 min from 50 to 90% B; 18 to 26 min from 90 to 99% B; 26 to 30 min at 99% B; 30 to 30.1 min from 99 to 30% B; 30.1 to 35 min at 30% B. The different theoretical *m/z* values of [M + NH_4_^+^] and [M - H^+^] ions are given in Table S5. The resulting data were analyzed and processed by the software Agilent MassHunter Qualitative Analysis 10.0 for the identification and the visualization of peaks/metabolites. The quantification was performed by Agilent MassHunter Quantitative Analysis software (Agilent Technologies).

### Proteomic analysis

Forty micrograms of membrane preparations were resuspended in 46 μl of a solution containing 5% SDS, 50 mM triethylammonium bicarbonate buffer pH 8.5, and 10 mM tris(2-carboxyethyl)phosphine. After 10 min at 95 °C, chloroacetamide was added to a final concentration of 20 mM followed by another 30 min at RT. Samples were then acidified by the addition of phosphoric acid to a final concentration of 2.5%, split into two parts and transferred onto a micro S-Trap column (Protifi LLC). Digestion was performed at 37 °C overnight using 1:100 of trypsin and LysC, either with or without 1 μl of PNGase F (500 Units, NEB).

Digested peptides were eluted in three steps using 40 μl each of 50 mM Tris pH 8.5, 0.2% formic acid, and 50% acetonitrile. The eluted peptides were dried down in a vacuum concentrator (SpeedVac, Thermo Fisher Scientific) and resuspended in 2% acetonitrile and 0.2% formic acid. Peptide concentration was determined by Pierce Quantitative Peptide Assay (Thermo Fisher Scientific). Peptides were directly loaded onto reversed-phase trap-column (Acclaim PepMap 100, Thermo Fisher Scientific) and eluted in backflush mode. Peptide separation was performed using a reversed-phase analytical column (EasySpray, 0.075 × 250 mm, ThermoFisher Scientific) with a linear gradient of 4% to 27.5% solvent B (0.1% formic acid in 98% acetonitrile) with solvent A (0.1% formic acid) for 100 min, 27.5% to 40% solvent B for 10 min, 40% to 95% solvent B for 10 min at a constant flow rate of 300 nl/min on a Vanquish Neo HPLC system. The peptides were analyzed by an Orbitrap Fusion Lumos tribrid mass spectrometer with an electrospray ionization source (ThermoFisher Scientific) coupled online to the nano-LC and operated in positive polarity. Peptides were detected in the Orbitrap at a resolution of 120,000. Peptides were selected for MS/MS using the HCD setting at 30; ion fragments were detected in the Orbitrap at a resolution of 30,000. A data-dependent procedure was used with a cycle time of 3 s alternating between one MS scan and multiple MS/MS scans for ions above a threshold ion count of 38,000 in the MS survey scan with 30 s dynamic exclusion. The electrospray voltage applied was 2.1 kV. MS1 spectra were obtained with an AGC target of 400,000 and a maximum injection time of 100 ms. MS2 spectra were acquired with an AGC target of 100,000 and an automatic adjustment of the maximum injection time, ms. The *m/z* scan range was 350 to 1800 for MS and automatically adjusted for MS/MS scans according to the precursor ion mass.

For samples without PNGase F treatment, a modified version with a second MS/MS step was used. When specific masses were observed in the first MS/MS step, EThcD fragmentation was triggered to facilitate the localization of the glycosylation site. This included m/z 204.0867 for HexNac, m/z 138.0545 for a HexNAc fragment, m/z 366.1396 for HexNac-Hex, m/z 292.102 for sialic acid, m/z 163.06 for Hex, and m/z 454.16 for HexNeuAc. The supplemental activation energy in EThcD was set to 30%, and ion fragments were detected in the Orbitrap at a resolution of 30,000, with an AGC target of 300,000, a maximum injection time of 250 ms, and an *m/z* scan range from 150 to 2000.

The resulting MS/MS data were processed using the Sequest HT search engine within Proteome Discoverer 2.5 SP1 against a *C. griseus* protein database obtained from Uniprot (sp_canonical TaxID=10029) with the addition of the human DHRSX sequence. Trypsin was specified as cleavage enzyme allowing up to two missed cleavages, four modifications per peptide, and up to seven charges. The mass error was set to 20 ppm for precursor ions and 0.05 Da for fragment ions. Oxidation on Met (+15.995 Da), Hex(1)HexNAc(2) (+568.212 Da), Hex(2)HexNAc(2) (+730.264 Da), Hex(3)HexNAc(2) (+892.317 Da), Hex(4)HexNAc(2) (+1054.370 Da), Hex(5)HexNAc(2) (+1216.423 Da), Hex(6)HexNAc(2) (+1378.476 Da), Hex(7)HexNAc(2) (+1540.529 Da), Hex(8)HexNAc(2) (+1702.581 Da), Hex(9)HexNAc(2) (+1864.634 Da), Hex(10)HexNAc(2) (+2026.687 Da), Asn->Asp (+0.984 Da) on asparagine and methionine loss (−131.040 Da) on the N-terminus of the protein and peptides were considered as variable modifications accordingly, whereas carbamidomethylation of cysteine was considered as a fixed modification. The false discovery rate was assessed using Percolator and thresholds for the identification of proteins, peptides, and modification sites were specified at 1%. Label-free quantification of peptides is based on the precursor ion intensity. Signals were normalized to the sum of all signals within each individual sample. Protein abundances were calculated as the sum of the abundances of unmodified peptides. To facilitate visualization in a heat map in Graphpad Prism, data were normalized to the 70th percentile of the abundance of the same peptide within the different samples.

### Long-read sequencing and assembly

High molecular weight genomic DNA from Pro-5, Lec5, and Lec9 CHO cells was extracted using the Monarch HMW DNA Extraction Kit for Cells & Blood (NEB, T3050L). Resulting gDNA was then sheared by passing each sample 25 times through a 26G needle. Average fragment lengths of 40 to 50 kbp were confirmed using a fragment analyzer (Advanced Analytical) with an HS Large Fragment 50 Kb analysis kit (Agilent, DNF-464-33). Samples were prepared for ONT long-read sequencing using a Ligation Sequencing Kit XL V14 (ONT, SQK-LSK114-XL) before loading of 1 μg DNA onto an R10.4.1 PromethION Flow Cell (ONT, FLO-PRO114M). Run quality control was performed using ONT’s MinKNOW Core 5.4.3. Resulting raw data was base called using Dorado 0.1.1 with the super accurate model. Raw statistics were obtained using NanoPlot 1.41.0 (40). Assembly was performed using flye 2.9.2 (41) and polished with racon 1.5.0 (42). Assembly quality was assessed with QUAST 5.2.0 (43). For mapping of flye-derived *de novo* assembly contigs or consensus sequences to the PICRH genome, Minimap 2.2.25 (44) was used for mapping of ONT reads and flye-derived *de novo* assembly contigs to the PICRH genome, with the models map-ont and asm20, respectively. Coverage was computed using Mosdepth 0.3.5 (45) and compiled using RStudio v4.3.2. Coverage and assembly data was analyzed and/or visualized using Integrative Genomics Viewer 2.17.1 (46), Ribbon (47), and NCBI’s Genome Workbench v4.9.1.

### Overexpression and Ni-NTA agarose purification of chDHRSX in *Escherichia coli*

pET15b plasmids containing the cDNA sequence of *chDHRSX* with a 6 × His tag appended to the N terminus were initially transformed into XL1 blue chemically competent *Escherichia coli* (Life technologies, ref: C404003). Clones were selected and grown to prepare minipreps. Vectors were then transformed into BL21(DE3). *E. coli* colonies were selected according to ampicillin resistance and grown in 10 ml LB medium (200 μg/ml ampicillin) overnight at 37 °C and 200 rpm before dilution in 200 ml LB medium (200 μg/ml ampicillin) and further growth until an A_600_ of 0.5 was reached. IPTG was then added to a final concentration of 1 mmol/L and the culture was grown for a further 20 h at 20 °C and 200 rpm before centrifugation at 4000*g* for 20 min to harvest cells. Native purification of 6 × His-tagged chDHRSX was performed using the AKTA purifier 900 series (GE Healthcare) by using a Ni^2+^-resin column (HisTrap-HP 1 ml, GE Healthcare) as described previously (48).

Protein concentration in the purified preparations was estimated by measuring A_280_ and computing the concentration from the expected extinction coefficient (ε = 29,450 M^−1^ cm^−1^) on the basis of the amino acid sequence (Protparam tool, at https://web.expasy.org/protparam/).

### Statistical analysis

All analyses were carried out *via* GraphPad Prism version 10.1 for Mac (GraphPad Software). Statistical analysis was performed by a student’s *t* test or one-way ANOVA followed by Tukey’s multiple comparisons test.

## Data availability

Mass spectrometry proteomics data have been deposited to the ProteomeXchange Consortium *via* the PRIDE partner repository with the dataset identifiers PXD052706 and 10.6019/PXD052706. ONT long-read sequencing data has been deposited to the NCBI Sequence Read Archive under the BioProject accession number PRJNA1120760.

## Supporting information

This article contains supporting information (46).

## Conflict of interest

The authors declare that they have no conflicts of interest with the contents of this article.
